# Topical Glucocorticosteroids for Proactive Therapy of Acute Radiation-Induced Skin Injury in Head and Neck Cancer: A Systematic Review and Meta-Analysis with Trial Sequential Analysis

**DOI:** 10.3390/biomedicines14040942

**Published:** 2026-04-21

**Authors:** Paweł Głuszak, Julia Woźna, Andrzej Bałoniak, Jakub Pazdrowski, Jan Stępka, Joanna Kaźmierska, Aleksandra Dańczak-Pazdrowska

**Affiliations:** 1 Department of Dermatology, Poznan University of Medical Sciences, 60-355 Poznan, Poland; 2 Doctoral School, Poznan University of Medical Sciences, 60-812 Poznan, Poland; 3 Department of Dermatology and Venereology, Poznan University of Medical Sciences, 60-355 Poznan, Poland; 4 Department of General Orthopaedics, Musculoskeletal Oncology, and Trauma Surgery, Poznan University of Medical Sciences, 60-355 Poznan, Poland; 5 Department of Head and Neck Surgery, Poznan University of Medical Sciences, 60-355 Poznan, Poland; 6 Department of Head and Neck Surgery, Greater Poland Cancer Centre, 61-866 Poznan, Poland; 7 Department of Electroradiology, Poznan University of Medical Sciences, 60-355 Poznan, Poland; 8 Department of Radiotherapy II, Greater Poland Cancer Centre, 61-866 Poznan, Poland

**Keywords:** radiation dermatitis, proactive therapy, radiodermatitis

## Abstract

**Background:** Acute radiation-induced skin injury (aRISI) is one of the most frequent adverse effects of radiotherapy (RT) in patients with head and neck cancer (HNC) and may compromise treatment delivery and quality of life. Topical glucocorticosteroids (GCS) are commonly used in clinical practice for aRISI management; however, evidence supporting their proactive use remains inconsistent. This systematic review and meta-analysis aimed to evaluate the efficacy and safety of proactive topical GCS therapy during RT for HNC. **Methods:** A systematic search of PubMed, Embase, and the Cochrane Library was conducted from database inception to July 2025 in accordance with PRISMA 2020 guidelines. Randomized controlled trials comparing topical GCS with placebo or standard skin care in adult patients undergoing curative RT or RChT for HNC were included. The primary outcomes were incidence of clinically significant aRISI (grade ≥ 2) and severe aRISI (grade ≥ 3), assessed using validated grading systems (RTOG or CTCAE). Random-effects meta-analyses were performed to calculate pooled risk ratios (RRs) with 95% confidence intervals (CIs). Risk of bias was assessed using the Cochrane RoB 2 tool. **Results:** Three randomized controlled trials comprising 459 patients were included. Proactive topical GCS did not significantly reduce the pooled incidence of grade ≥ 2 aRISI compared with placebo or standard skin care (RR 0.87, 95% CI 0.60–1.27). Similarly, no statistically significant reduction in grade ≥ 3 aRISI was observed in pooled analysis (RR 0.81, 95% CI 0.22–3.06). Qualitative synthesis of secondary outcomes reported in individual trials suggested potential benefits of topical GCS, including delayed onset or slower progression of aRISI, and, in one large double-blind study, a reduced risk of severe reactions. No increase in treatment-related adverse events was observed in any included trial. **Conclusions:** Proactive topical GCS do not significantly reduce the overall incidence of aRISI in pooled analysis. Individual studies showed trend towards delayed onset, slower progression, and reduced severe aRISI without compromising safety. These findings support the judicious use of topical GCS as part of proactive supportive care in HNC RT, while highlighting the need for larger, standardized trials to define optimal regimens and patient selection.

## 1. Introduction

Head and neck cancer (HNC) represents a substantial and growing global health burden [1,2,3,4,5]. Its incidence continues to rise worldwide, driven not only by population aging but also by increased tobacco use in developing countries and the expanding prevalence of human papillomavirus (HPV)-associated malignancies in Western societies [2,3]. Radiotherapy (RT) remains a cornerstone of both curative and palliative treatment for early-stage and locally advanced HNC [6,7]. As a function-preserving and minimally invasive modality, RT is frequently used alone or in combination with surgery and/or systemic therapy. However, despite its therapeutic efficacy, RT is commonly associated with treatment-related adverse effects, among which acute radiation-induced skin injury (aRISI) is the most frequent, affecting up to 100% of patients undergoing RT for HNC [8]. This high incidence is probably due to exposome, including environmental and lifestyle factors such as ultraviolet exposure, air pollution, and tobacco use [8,9]. Clinically significant aRISI may lead to treatment interruptions or delays and can markedly impair patients’ quality of life.

aRISI, also referred to as radiation dermatitis or radiodermatitis, is an inflammatory cutaneous reaction characterized by erythema, dry or moist desquamation, pain, and, in severe cases, secondary infection or ulceration [8,10,11,12]. Main two scales used to assess aRISI severity are Radiation Therapy Oncology Group (RTOG) scale and Common Terminology Criteria for Adverse Events (CTCAE) scale [8,11]. Although the pathophysiology of aRISI has not been fully elucidated, accumulating evidence indicates that radiation-induced DNA damage and reactive oxygen species (ROS) generation disrupt cellular homeostasis and initiate a cascade of inflammatory responses [13,14,15,16]. These processes are mediated by the upregulation of multiple pro-inflammatory cytokines and adhesion molecules, including eotaxin, ICAM-1, interleukins (IL-1, IL-3, IL-5, IL-6, IL-8), and tumor necrosis factor alpha (TNF-α), ultimately leading to epidermal injury and impaired barrier function [16].

Topical glucocorticosteroids (GCS) directly target these inflammatory pathways and therefore represent a biologically plausible strategy for the prevention and treatment of aRISI [17]. Their anti-inflammatory effects are mediated through suppression of multiple immune cell populations and downregulation of cytokines such as IL-1α, IL-1β, IL-2, TNF-α, and granulocyte–macrophage colony-stimulating factor (GM-CSF) [18]. In clinical practice, management of aRISI typically includes proper skin care, namely the use of emollients, gentle cleansing products, UV protection, gentle clothing, as well as smoking cessation. Moreover, topical GCS, and specialized wound care, depending on the severity of skin reactions, are employed [8,13]. Recent Multinational Association of Supportive Care in Cancer (MASCC) guidelines published in 2023 recommend topical GCS as generally safe and effective for both prophylaxis and treatment of aRISI; however, they also emphasize the need for further high-quality evidence, as current data remain insufficient and heterogeneous [10]. Consequently, substantial variability persists in aRISI management across institutions, with approaches often dependent on local protocols and clinical experience rather than robust evidence [8,10,12].

In recent years, increasing attention has been directed toward proactive therapy, which involves the early initiation of topical agents, including GCS, before or at the onset of RT, with the aim of mitigating inflammation before clinically significant skin injury develops [10]. While several systematic reviews and meta-analyses have evaluated prophylactic interventions for radiation dermatitis, the results remain inconsistent [19,20]. Some meta-analyses have suggested that no proactive intervention is superior to standard skin care, whereas others have identified potential benefits of selected agents, including topical GCS. However, many of these analyses included heterogeneous cancer populations, mixed anatomical sites, and interventions that are not routinely used in clinical practice, limiting their applicability to patients with HNC.

Taken together, the existing evidence remains conflicting, and robust data specifically addressing proactive topical GCS use in patients undergoing HNC RT are limited. Given the high incidence and clinical impact of aRISI in this population, clarification of the role of topical GCS is urgently needed. The aim of this systematic review and meta-analysis was to evaluate the efficacy and safety of proactive topical GCS therapy during RT for HNC, with a focus on reducing the incidence and severity of aRISI. By synthesizing the available randomized evidence, this study seeks to provide a clearer evidence base for the rational integration of topical GCS into supportive care protocols for patients undergoing HNC RT.

## 2. Materials and Methods

This research followed the PRISMA 2020 standards and the Cochrane Handbook for Systematic Reviews and Meta-Analyses [21,22]. The systematic review was carried out in line with the PRISMA 2020 checklist and was registered in advance with PROSPERO (registration number CRD420251031606) [23].

### 2.1. Eligibility Criteria

Eligibility criteria were defined according to the PICO framework. The study population (P) included adult patients with histologically confirmed head and neck carcinoma (HNC) undergoing definitive or postoperative RT or RChT with curative intent. The intervention (I) consisted of topical corticosteroid application (including betamethasone valerate or other topical GCS) administered during RT, either prophylactically or at early stages of radiation dermatitis. The comparator (C) included standard skin care, placebo, or no topical steroid application, depending on study design. Studies were required to report at least one of the predefined outcomes (O), including incidence and severity of acute radiation dermatitis, particularly grade ≥ 2 or grade ≥ 3 toxicity, assessed using validated grading systems such as the Radiation Therapy Oncology Group (RTOG) or Common Terminology Criteria for Adverse Events (CTCAE) scales. Exclusion criteria comprised case reports, non-comparative studies, reviews, non-English publications, and studies involving non–head and neck malignancies or palliative RT.

### 2.2. Search Strategy

A thorough literature review was performed using PubMed (MEDLINE), the Cochrane Library, and Embase, covering the period from their inception until 7 July 2025. The search strategy employed a combination of thesaurus terms utilized by the databases (such as MeSH terms) and free terms. The search utilized, among others, terms such as “radiodermatitis”, “topical corticosteroid” and “head and neck cancer” which were combined with Boolean operators (“AND”, “OR”). The detailed search methodology can be found in Supplementary Material S1.

### 2.3. Data Collection Process and Extracted Variables

Two independent reviewers were responsible for selecting studies. After removing duplicates with Zotero (version 7), they screened titles and abstracts before proceeding to a full-text review. Any disagreements were settled through consensus or by consulting a third reviewer. The data collected from each qualifying study included the first author’s name, publication year, country, study design, total sample size, and participant characteristics such as age and gender distribution.

### 2.4. Risk of Bias Assessment

Two independent reviewers evaluated the risk of bias using the Cochrane Risk of Bias 2 (RoB 2) tool for randomized controlled trials [24]. Any disagreements were settled through discussion or by involving a third reviewer for adjudication. Publication bias was assessed by employing funnel plots to detect potential asymmetry and outlier studies.

### 2.5. Statistical Analysis

All statistical analyses were conducted using R software (v0.9.5.10 Beta). For binary variables, the incidence numbers were input, and pooled risk ratios (RRs) with 95% confidence intervals (CIs) were determined through a Mantel-Haenszel random-effects model (Paul–Mandel estimator of τ^2^) with Hartung-Knap adjustment for the confidence intervals. Heterogeneity was evaluated using Cochran’s Q test (with *p* < 0.10 indicating significance) and the I^2^ statistic (where I^2^ > 50% suggests substantial heterogeneity). Leave-one-out sensitivity analyses were carried out to assess the impact of each individual study on the pooled estimates. Trial sequential analysis (TSA) was performed using TSA software (Copenhagen Trial Unit, Copenhagen, Denmark), with a two-sided α of 5%, power of 80%, and O’Brien–Fleming alpha-spending boundaries, to assess the risk of random error and determine whether the available evidence is sufficient to draw reliable conclusions [25].

## 3. Results

### 3.1. Search Results

**Figure 1:**
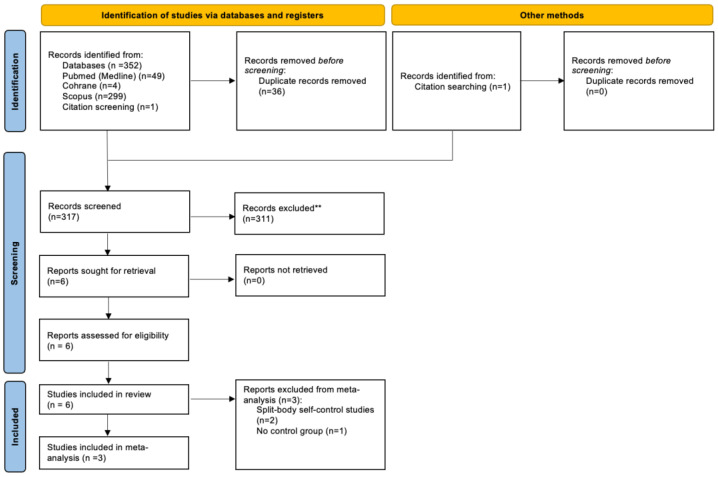
Flow diagram showing the identification and selection process for studies. ** Excluded due to no relevance or improper study design.

The systematic literature search identified records through database searching and additional sources. After removal of duplicates, titles and abstracts were screened for eligibility. Full-text articles were subsequently assessed, and three randomized controlled trials comparing topical GCS versus placebo or standard of care in patients undergoing RT were included in the final qualitative and quantitative synthesis [26,27,28]. The study selection process is summarized in the PRISMA flow diagram in Figure 1.

### 3.2. Study Characteristics

The meta-analysis included three randomized controlled trials, published between 2020 and 2021, enrolling a total of 459 patients undergoing RT [26,27,28]. Across the included studies, 228 patients received topical GCS and 231 patients received placebo or standard of care. All studies evaluated the effectiveness of topical GCS for the prevention or reduction of radiodermatitis, with outcomes reported as either overall radiodermatitis or more severe radiodermatitis. Detailed study characteristics are presented in Table 1.

### 3.3. Risk of Bias Assessment

**Figure 2:**
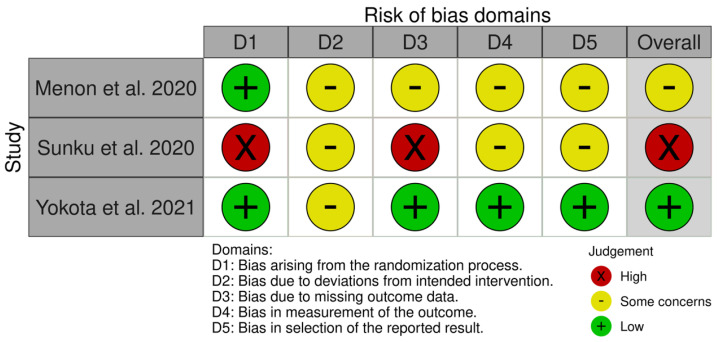
Evaluation of Risk of Bias using the Cochrane Risk of Bias 2 (RoB2) tool. The risk of bias across domains was graphically represented in green color (low risk of bias), yellow (moderate risk of bias), and red (high risk of bias) [26,27,28].

Risk of bias was assessed using the Cochrane Risk of Bias 2 (RoB 2) tool across five domains (Figure 2). Regarding bias arising from the randomization process (Domain 1), two studies were judged to be at low risk, while one study was rated as high risk due to insufficient reporting of sequence generation and allocation concealment. For deviations from intended interventions (Domain 2), all studies raised some concerns, mainly because of limited information on blinding of participants and treating personnel and the absence of detailed adherence reporting. In Domain 3 (missing outcome data), one study was assessed as low risk, whereas another was classified as high risk owing to incomplete outcome data and unclear handling of attrition; the remaining study raised some concerns. Assessment of bias in outcome measurement (Domain 4) showed one study at low risk, while the remaining studies raised some concerns, largely due to insufficient reporting on blinding of outcome assessors, despite the use of standardized radiodermatitis grading systems. For selective reporting (Domain 5), one study was judged at low risk, whereas two studies raised some concerns, as no publicly available protocols or prespecified analysis plans were provided. Overall, one study was rated as having a low risk of bias, one as having some concerns, and one as having a high overall risk of bias, which should be considered when interpreting the pooled effect estimates.

### 3.4. Meta-Analysis Results

#### 3.4.1. Severe aRISI

**Figure 3:**
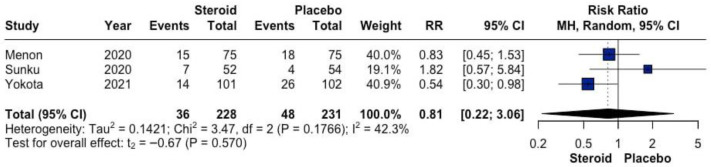
Forrest plot graphically representing the meta-analysis on the effect of topical GCS compared with placebo or standard of care on severe aRISI. Diamond, depicted in the dark color, indicate the overall effect size—pooled RRs were used as effect size metric—jointly with their corresponding 95% confidence intervals (95% CIs) [26,27,28].

The effect of topical GCS compared with placebo or standard of care on severe aRISI is presented in Figure 3. Across the three included randomized studies, 36 events occurred among 228 patients in the GCS group (15.8%), compared with 48 events among 231 patients in the placebo or standard of care group (20.8%). Pooled analysis using a random-effects model demonstrated no statistically significant difference between groups (RR 0.81, 95% CI 0.22–3.06; *p* = 0.57). Moderate heterogeneity was observed (I^2^ = 42.3%).

#### 3.4.2. Overall aRISI

**Figure 4:**
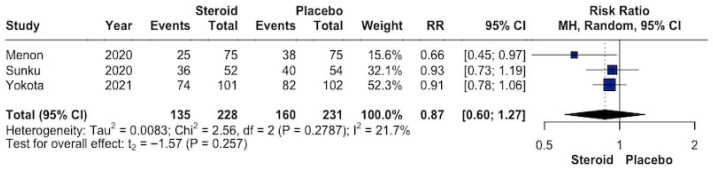
Forrest plot graphically representing the meta-analysis on the effect of topical GCS compared with placebo or standard of care on overall aRISI. Diamond, depicted in the dark color, indicate the overall effect size—pooled RRs were used as effect size metric—jointly with their corresponding 95% confidence intervals (95% CIs) [26,27,28].

The pooled effect of topical GCS versus placebo or standard of care on overall aRISI is shown in Figure 4. In total, 135 of 228 patients receiving topical GCS (59.2%) and 160 of 231 patients receiving placebo or standard of care (69.3%) experienced aRISI. The meta-analysis showed no significant reduction in risk associated with steroid use (RR 0.87, 95% CI 0.60–1.27; *p* = 0.26). Low heterogeneity was detected across studies (I^2^ = 21.7%).

#### 3.4.3. Leave-One-Out Analysis

**Figure 5:**
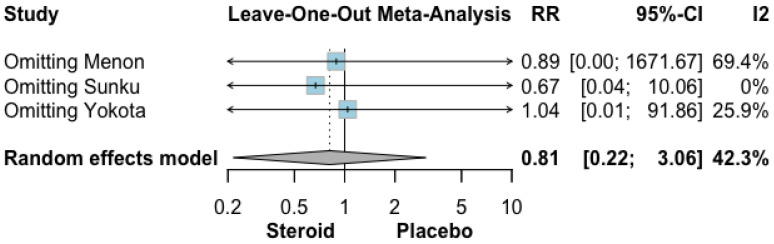
Leave-one-out analysis results of severe aRISI [26,27,28].

**Figure 6:**
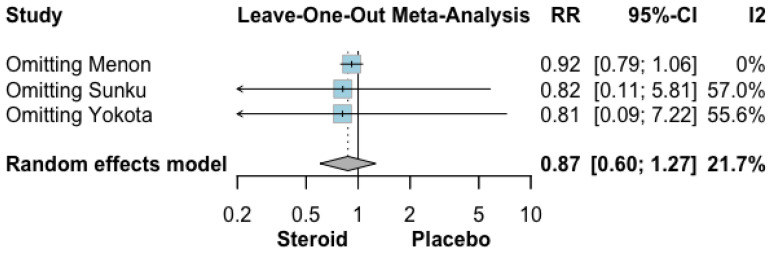
Leave-one-out analysis results of overall aRISI [26,27,28].

In the leave-one-out analysis (Figure 5 and Figure 6), omitting the study by Menon et al. reduced heterogeneity to 0% while leaving the pooled estimate for overall aRISI essentially unchanged [26]. Similarly, omitting the study by Sunku et al. reduced heterogeneity to 0% without materially affecting the pooled estimate for severe aRISI [27].

#### 3.4.4. Publication Bias

**Figure 7:**
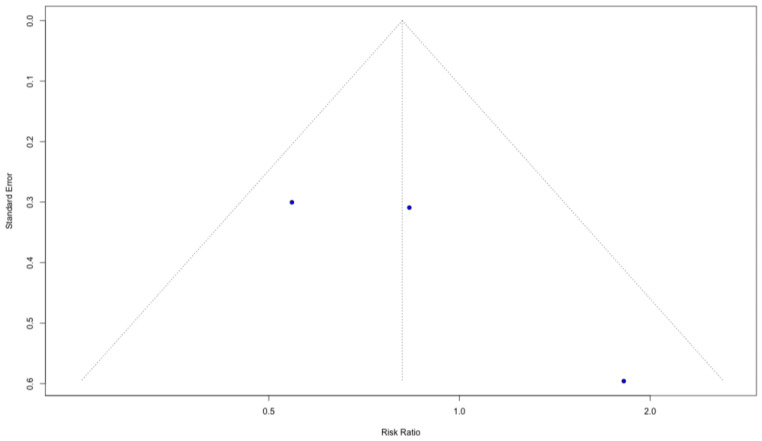
Funnel plot for severe aRISI.

**Figure 8:**
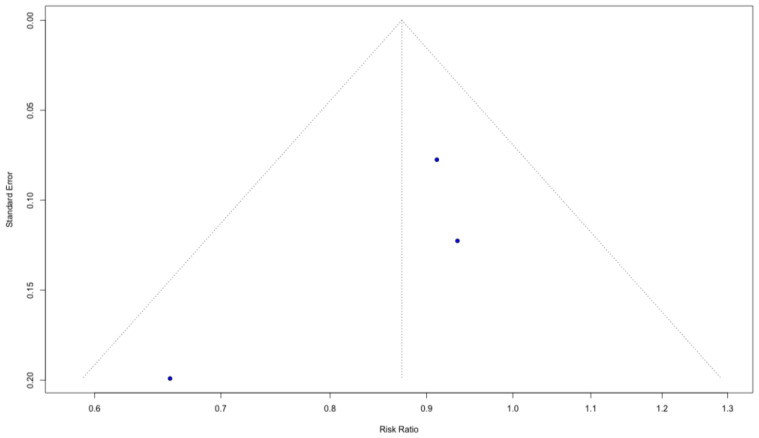
Funnel plot for overall aRISI.

Visual inspection of funnel plots for both outcomes did not identify any clear outliers or studies exerting a disproportionate influence on the pooled estimates (Figure 7 and Figure 8). Although the small number of included trials limits the interpretability of funnel plot asymmetry, the observed distribution does not suggest that the overall results are driven by single extreme effects.

#### 3.4.5. Trail Sequential Analysis

**Figure 9:**
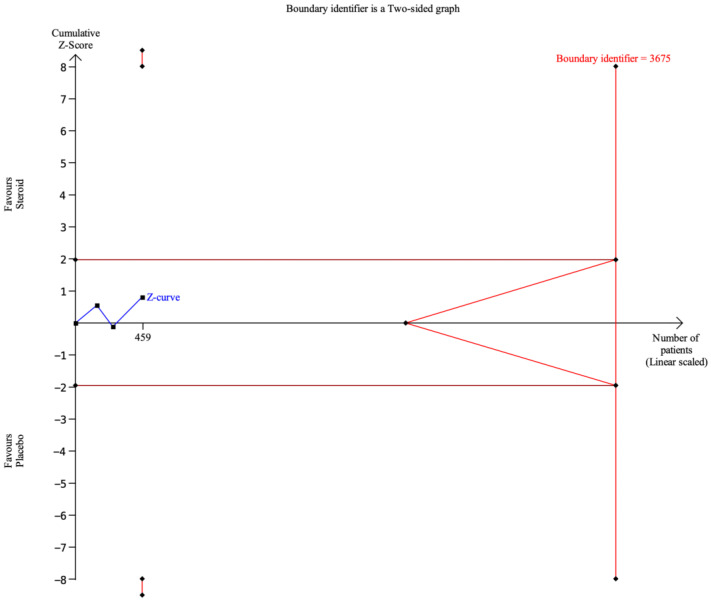
Trial sequential analysis of severe aRISI. X-axis: the number of patients randomized; Y-axis: the cumulative Z-score; horizontal red lines: conventional boundaries (upper for benefit, Z-score = 1.96; lower for harm, Z-score = −1.96; two-sided P = 0.05); red lines indicating trial sequential monitoring boundaries; blue line with black square fill icons: cumulative Z-curve; vertical red line: required information size.

For the outcome of severe aRISI, the cumulative Z-curve did not cross the conventional boundary for statistical significance or the trial sequential monitoring boundaries and did not enter the futility area (Figure 9). The required information size was estimated at 3675 patients, whereas the currently accumulated sample size was 459 patients.

**Figure 10:**
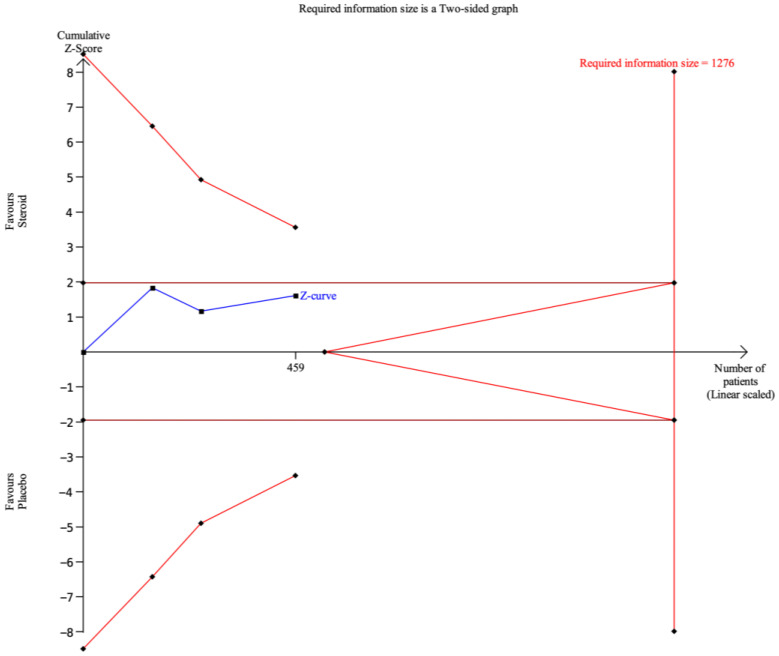
Trial sequential analysis of overall aRISI. X-axis: the number of patients randomized; Y-axis: the cumulative Z-score; horizontal red lines: conventional boundaries (upper for benefit, Z-score = 1.96; lower for harm, Z-score = −1.96; two-sided P = 0.05); sloping red lines: trial sequential monitoring boundaries; blue line with black square fill icons: cumulative Z-curve; vertical red line: required information size.

For the outcome of overall aRISI, the cumulative Z-curve did not cross the conventional boundary for statistical significance or the trial sequential monitoring boundaries and did not enter the futility area (Figure 10). The required information size was estimated at 1276 patients, whereas the currently accumulated sample size was 459 patients. The cumulative Z-curve showed a trend toward statistical significance in favor of topical GCS.

## 4. Discussion

This systematic review and meta-analysis synthesized the available randomized evidence on the proactive use of topical GCS for the prevention and mitigation of aRISI in patients undergoing curative RT or RChT for HNC. Based on three randomized controlled trials enrolling a total of 459 patients, topical GCS did not significantly reduce the pooled incidence of clinically relevant aRISI (grade ≥ 2) compared with placebo or standard skin care. Additionally, the validity of the pooled evidence is limited, as only one study was assessed as having low risk of bias, while the remaining two trials presented either some concerns or high risk of bias. This further reduces confidence in the pooled estimates and reinforces the need for cautious interpretation. However, across individual studies, a trend toward clinical benefit was observed. In raw numbers, patients treated with topical GCS had less severe/overall aRISI than those receiving placebo or standard of care. However, this difference did not reach statistical significance in our analysis. It should be noted that, due to small number of studies eligible for quantitative synthesis, we adopted conservative statistical approach to ensure more transparent and robust results. Nevertheless, the leave-one-out analysis showed that excluding any single study did not meaningfully change the pooled effect. Likewise, visual inspection of the funnel plots did not reveal any clear outliers that would be likely to materially influence the overall results; however hence only 3 studies were included, funnel plots should be interpreted with caution. Importantly, excluding the study assessed as having a high risk of bias [27] also did not affect our findings. Taken together, these observations suggest that the overall direction of the effect appears consistent across studies, although firm conclusions remain limited by the small evidence base.

According to the TSA, the current body of evidence derived from quantitative synthesis is insufficient to draw definitive conclusions regarding the impact of topical GCS on aRISI outcomes. The cumulative Z-curve did not intersect the conventional or trial sequential monitoring boundaries, nor did it achieve the required information size. Importantly, the Z-curve also did not enter the futility area. This pattern suggests a potential risk of type II error (false-negative result), indicating that the lack of statistical significance may be attributed to inadequate statistical power rather than an actual absence of effect. Nevertheless the trajectory of the cumulative Z-curve aligns with the results of the conventional meta-analysis, suggesting that topical GCS do not exacerbate outcomes and may be associated with a potential reduction in overall aRISI. Importantly, synthesizing the currently available randomized evidence, even if limited, provides clinically meaningful information. In particular, the present analysis helps to clarify that the existing data do not support definitive conclusions regarding the routine use of topical GCS in this setting and that further adequately powered studies are required. From both a clinical and research perspective, defining the degree of uncertainty and quantifying the insufficiency of the evidence base—supported by TSA—represents valuable information in itself. It may help clinicians avoid overinterpretation of small individual studies and assist researchers in designing future trials with appropriate sample sizes and methodological rigor.

The timing of intervention appears to be one of the key determinants of therapeutic effect. In studies employing prophylactic GCS use from the initiation of RT, treatment was associated with delayed onset and slower progression of aRISI, with fewer early grade 1-2 reactions [26,27]. In contrast, the study initiating GCS reactively, meaning at the onset of grade 1 aRISI or at a predefined dose threshold—demonstrated a significant reduction in progression to grade ≥ 3 toxicity but no effect on overall aRISI incidence [28]. This pattern suggests that prophylactic application may modulate early inflammatory processes, whereas reactive use may be more effective in preventing escalation to severe tissue injury. These findings indicate that proactive strategies may need to be tailored to specific clinical goals and patient risk factors.

Across all included studies, topical GCS were well tolerated, with no increase in adverse effects such as infection, delayed wound healing, or treatment interruptions. This is clinically relevant, as concerns regarding steroid-related skin atrophy, telangiectasia, or secondary infection often limit their use, particularly in the head and neck region [29]. The favorable safety profile observed in this analysis supports the short-term use of topical GCS during RT when appropriately monitored, even in this anatomically sensitive area.

Beyond intervention-specific effects, the importance of identifying effective proactive therapy regimens is underscored by the multifactorial nature of aRISI risk in patients undergoing HNC RT. The severity of aRISI is influenced by numerous treatment-related factors, including cumulative skin dose, fractionation, irradiated volume, RT technique, use of bolus material, and concurrent systemic therapy, as well as patient-related factors such as smoking status, nutritional status, comorbidities (e.g., diabetes or vascular disease), baseline skin condition, and environmental exposures [8,30,31,32,33,34,35,36,37,38,39]. This substantial individual variability may attenuate observable treatment effects when prophylaxis is applied uniformly across heterogeneous populations. Consequently, future supportive care strategies should emphasize both the identification of high-risk patients and the development of standardized, evidence-based proactive protocols that allow for risk-adapted interventions while maintaining clinical feasibility and reproducibility across treatment centers.

Several studies identified during the literature search were excluded from quantitative synthesis due to methodological constraints but provide valuable context. Fazeel et al. (2022) conducted a prospective, open-label, randomized controlled trial in India comparing the efficacy of mometasone furoate 0.1% cream versus betamethasone valerate 0.1% cream for the management of aRISI in 123 patients with HNSCC undergoing curative RT [40]. Both treatments were applied daily throughout RT, and outcomes were assessed using RTOG grading as well as patient-reported pain and itching scores. The study demonstrated that mometasone was significantly more effective than betamethasone, with a higher proportion of patients achieving improvement to RTOG grade 1 (62.9% vs. 29.5%, *p* < 0.05), as well as greater reductions in pain and pruritus. No treatment-related adverse effects were observed, and both topical GCS were more effective at skin doses below 6000 cGy, suggesting a dose-dependent response. This study was excluded from the meta-analysis because it lacked a placebo or standard-care control group and compared two active topical GCS. Sperduti et al. (2006) conducted a prospective feasibility study using an internal control (split-neck) design to evaluate the effect of 1% hydrocortisone cream on aRISI in patients receiving radical RT for HNC [41]. Twenty patients were planned for enrollment, but the study was terminated early after only 12 patients were accrued, as the hydrocortisone-treated side consistently demonstrated higher skin toxicity scores compared with the untreated control side, as measured by both the RTOG and Radiation-Induced Skin Reaction Assessment Scale (RISRAS). The increased severity of erythema and dry desquamation on the treated side was confirmed by blinded assessors, photographic documentation, and patient-reported symptoms, leading to early discontinuation of the intervention. The study concluded that although the internal control methodology was feasible and reliable, the sample size was too small to draw definitive conclusions regarding efficacy, and hydrocortisone was unexpectedly associated with worsened aRISI. This study was excluded from the meta-analysis because it used a split-body internal control design without independent parallel treatment groups and was terminated early due to harm, precluding extraction of comparable binary outcome data for quantitative synthesis. Liao et al. (2019) conducted a randomized, prospective, self-controlled trial evaluating the efficacy of mometasone furoate 0.1% cream in reducing aRISI in 41 patients with HNSCC undergoing bilateral IMRT with identical neck doses [42]. One side of the neck was randomized to receive once-daily mometasone application from the first day of RT, while the contralateral side received no topical treatment. The study demonstrated a significant reduction in RTOG scores on the treated side compared with the control side, particularly when the skin radiation dose was below 6000 cGy, while no significant benefit was observed at higher doses. In addition, mometasone significantly reduced patient-reported pain and pruritus regardless of radiation dose, with no steroid-related adverse events reported. This study was excluded from the meta-analysis because it employed a split-body self-controlled design without independent parallel treatment groups.

The findings of this meta-analysis are broadly consistent with evidence from breast cancer RT, where topical GCS have been shown to reduce higher-grade aRISI while having less impact on mild skin reactions [19,43]. However, extrapolation to HNC is limited by fundamental anatomical and biological differences. The skin of the head and neck is thinner, more exposed to ultraviolet radiation, and more frequently affected by tobacco-related damage. In addition, patients with HNC often present with comorbidities and lifestyle factors that impair skin barrier function and wound healing [9]. These features may explain both the higher incidence of aRISI in this population and the variable response to topical interventions.

The use of topical GCS in the head and neck region remains controversial in dermatologic practice due to concerns about steroid-induced adverse effects, including rosacea-like dermatitis, skin atrophy, and telangiectasia [29]. Nevertheless, the absence of such complications in the included trials, combined with the short duration of use and clear symptomatic benefits, suggests that carefully selected GCS regimens may be safely incorporated into supportive care. Alternative anti-inflammatory approaches, such as topical calcineurin inhibitors, may represent promising safe and efficacious options, particularly for prolonged use; however, no randomized studies have evaluated these agents in the setting of aRISI to date [44,45,46,47].

The lack of statistically significant pooled benefit must be interpreted in the context of substantial clinical and methodological heterogeneity. Included trials differed in the type and potency of GCS used (difluprednate is more potent than betamethasone), dosing frequency (once versus twice daily), timing of initiation, duration of treatment, and comparator regimens. In addition, RT techniques varied across studies, including conventional techniques and intensity-modulated radiotherapy (IMRT), which are known to differ in skin dose distribution and toxicity profiles [48,49]. The use of concurrent chemotherapy, particularly cisplatin, also varied and likely contributed to differences in aRISI severity [50,51,52,53]. Outcome assessment methods further differed, with two trials using the RTOG scale and one employing CTCAE grading with centralized photographic review, potentially introducing classification heterogeneity [11]. Moreover, there is a key source of clinical heterogeneity is the timing of topical GCS initiation. Two trials started GCS from day 1 of RT (true prophylaxis), whereas Yokota et al. initiated treatment at the onset of grade 1 aRISI or at a cumulative dose threshold (≥30 Gy), representing an early/interceptive strategy rather than true prophylaxis. This difference may partly explain why pooled analyses did not show a clear reduction in overall aRISI incidence, while signals of benefit were observed for progression to severe (grade ≥ 3) aRISI in Yokota et al. [28].

Another important limitation is the restricted geographic representation of the included studies. All analyzed trials were conducted in Asian populations (India and Japan), which may limit the generalizability of the findings to other populations. Differences in patient characteristics, skin types, environmental exposures, and clinical practice patterns may influence both the incidence and severity of aRISI as well as response to topical interventions.

While these limitations warrant caution, the present study provides a structured synthesis of the highest level of available evidence and incorporates trial sequential analysis, which strengthens the interpretation by quantifying the degree of uncertainty and the need for further research.

Despite increasing research interest, the field of proactive therapy for aRISI remains fragmented. Previous meta-analyses have evaluated a wide range of interventions, including barrier films, dressings, natural compounds, laser therapy, and topical agents, with inconsistent results. Current MASCC guidelines emphasize barrier protection and individualized supportive care but acknowledge the limited quality of available evidence [10,54]. Our findings support this cautious stance and underscore the need for high-quality, head and neck specific trials.

Future research should prioritize large, multicenter, double-blind randomized controlled trials with standardized skin care protocols, uniform outcome definitions, and stratification by RT technique and concurrent chemotherapy. Comparative studies evaluating GCS potency, dosing frequency, and duration, as well as direct comparisons between prophylactic and reactive strategies, are particularly needed. Integration of patient-reported outcomes and quality-of-life measures will be essential to capture the full clinical impact of aRISI and its management.

## 5. Conclusions

Pooled estimates from three randomized trials did not demonstrate a statistically significant reduction in the incidence of grade ≥ 2 or grade ≥ 3 aRISI with proactive topical GCS. However, both the direction of effect in the meta-analysis and the trial sequential analysis suggest a consistent trend toward benefit. Importantly, the TSA indicated that the required information size has not been reached and that the cumulative Z-curve has not entered the futility area, highlighting a potential risk of type II error (false-negative result).

Qualitative synthesis of individual trials further supports this signal, suggesting delayed onset and/or slower progression of aRISI and, in one large double-blind trial, a reduction in severe reactions. No increase in treatment-related adverse events was observed.

Taken together, these findings suggest that proactive topical GCS may be beneficial in this setting, although current evidence remains insufficient to draw definitive conclusions. Larger, adequately powered and standardized trials are required to confirm these effects and define optimal use.

## Figures and Tables

**Table 1:** Summarized characteristics of the studies. Abbreviations: GCS—glucocorticosteroids; aRISI—acute radiation-induced skin injury; RT—radiotherapy.

Study	Study Design	Sample Size	Radiation Technique and Dose	Concurrent Chemotherapy	Clinical Scale Employed	Intervention	Timing of Application	Comparator	Primary Skin Toxicity Outcomes
Menon et al., 2020 [26]	Phase 3, randomized, open-label, single-center	150 randomized (75 GCS, 75 control); 121 completed (61 GCS, 60 control)	2-dimensional conventional RT or IMRT, total dose: 60-6 Gy, 2 Gy/dose	Concurrent chemotherapy in 49.3%, usually cisplatin 100 mg/m^2^ every 3 weeks	RTOG, RISRAS	Betamethasone valerate 0.1% cream, once daily	From first day of RT, continued during and until 2 weeks after RT	Basic skin care	The frequency of grade ≥ 2 aRISI was significantly reduced; however, the occurrence of grade ≥ 3 aRISI was not significantly reduced.
Sunku et al., 2020 [27]	Prospective, randomized, open-label	106 randomized (52 GCS, 54 control); 85 completed (44 GCS, 41 control)	2-dimensional conventional RT, total dose: 66-70 Gy, 2 Gy/dose	Concurrent chemotherapy in 65%, usually cisplatin or carboplatin	RTOG	Betamethasone valerate 0.1% cream, twice daily	From day 1 of RT (or ≤day 3), continued throughout RT	Basic skin care	The onset of aRISI was delayed and progression was slower in the GCS group, with fewer early grade 1–2 reactions; no difference was observed in grade 3–4 aRISI or in time to healing.
Yokota et al., 2021 [28]	Phase 3, multicenter, randomized, double-blind, placebo-controlled	211 randomized (101 GCS, 102 placebo); 195 completed (97 GCS, 98 control)	3-dimensional conventional RT or IMRT, total dose over 60 Gy, 2 Gy/dose	Concurrent chemotherapy in 100%, usually cisplatin in different dosing	CTCAE v 4.0	Difluprednate 0.05% ointment, at least once daily	Started when grade 1 dermatitis appeared or at 30 Gy, continued ≥2 weeks post-RT	Placebo (white vaseline ointment + identical basic skin care)	The frequency of grade ≥ 2 aRISI was not significantly reduced; however, the occurrence of grade ≥ 3 aRISI was significantly reduced in the GCS group, with no significant differences in adverse effects.

## Data Availability

Not applicable.

## Supplementary Materials

The following supporting information can be downloaded at: https://www.mdpi.com/article/10.3390/biomedicines14040942/s1, (S1) Detailed methodology search.

## Abbreviations

aRISI	acute radiation-induced skin injury
CI	confidence interval
CTCAE	Common Terminology Criteria for Adverse Events
DNA	deoxyribonucleic acid
GM-CSF	granulocyte–macrophage colony-stimulating factor
GCS	glucocorticosteroids
HNC	head and neck cancer
HNSCC	head and neck squamous cell carcinoma
HPV	human papillomavirus
ICAM-1	intercellular adhesion molecule 1
IL	interleukin
IMRT	intensity-modulated radiotherapy
MASCC	Multinational Association of Supportive Care in Cancer
MeSH	Medical Subject Headings
PICO	Population, Intervention, Comparator, Outcomes
PRISMA	Preferred Reporting Items for Systematic Reviews and Meta-Analyses
PROSPERO	International Prospective Register of Systematic Reviews
RoB 2	Risk of Bias 2 tool
ROBINS-I	Risk Of Bias In Non-randomized Studies of Interventions
ROS	reactive oxygen species
RR	risk ratio
RT	radiotherapy
RTOG	Radiation Therapy Oncology Group
RChT	radiochemotherapy
RISRAS	Radiation-Induced Skin Reaction Assessment Scale
TNF-α	tumor necrosis factor alpha
BMI	body mass index
UV	ultraviolet
