# Supplementary material for: Topical Glucocorticosteroids for Proactive Therapy of Acute Radiation-Induced Skin Injury in Head and Neck Cancer: A Systematic Review and Meta-Analysis with Trial Sequential Analysis
Source: Biomedicines. 2026 Apr 21;14(4):942. doi: 10.3390/biomedicines14040942 (PMC13113669; doi:10.3390/biomedicines14040942)
Supplement: Supplementary file 1 [file biomedicines-14-00942-s001.zip › biomedicines-4192511-supplementary.pdf]

Supplementary material S1

Research strategy – example for PubMed

```
(
 radiodermatitis
 OR "radiation dermatitis"
 OR "radiation-induced dermatitis"
 OR "radiation induced dermatitis"
 OR "radiation-induced skin injury"
 OR "radiation induced skin injury"
 OR "radiation-induced skin toxicity"
 OR "radiation induced skin toxicity"
 OR "radiation skin toxicity"
 OR "radiation-related skin toxicity"
 OR "acute radiation skin reaction*"
 OR "acute skin toxicity"
 OR "radiation skin reaction*"
)
AND
(
 "topical corticosteroid*"
 OR corticosteroid*
 OR "topical steroid*"
 OR steroid
 OR steroids
 OR hydrocortisone
 OR betamethasone
 OR mometasone
 OR topical
 OR ointment
 OR ointments
 OR cream
 OR creams
)
AND
(
 "head and neck cancer"
 OR "head and neck carcinoma"
 OR "head-and-neck cancer"
 OR "head and neck neoplasm*"
 OR "head and neck malignanc*"
 OR "head and neck squamous cell carcinoma"
 OR HNSCC
 OR "oropharyngeal cancer"
 OR "nasopharyngeal cancer"
 OR "laryngeal cancer"
 OR "hypopharyngeal cancer"
 OR "oral cavity cancer"
 OR "oral cancer"
 OR "oral squamous cell carcinoma"
 OR "salivary gland cancer"
)
```
